# Astaxanthin–Turmeric–Honeysuckle Extract Combination Attenuates DSS-Induced Colitis with Preservation of the Epithelial Barrier and Reduced NLRP3/Caspase-1/GSDMD Signaling

**DOI:** 10.3390/molecules31183303

**Published:** 2026-09-17

**Authors:** Zhongting Lv, Xuan Liu, Yong Pang, Jie Zhang, Li Ren

**Affiliations:** College of Food Science and Engineering, Jilin University, Changchun 130062, China

**Keywords:** dextran sulfate sodium, intestinal epithelial barrier, pyroptosis, NLRP3 inflammasome

## Abstract

Inflammatory bowel disease is characterized by recurrent mucosal inflammation, epithelial barrier disruption, and dysregulated cell death, highlighting the need for safe, multi-target interventions. Natural astaxanthin, renewably sourced from *Haematococcus pluvialis* or *Phaffia rhodozyma*, has shown greater tissue accumulation or antioxidant activity than synthetic astaxanthin in some preparations. Based on the complementary antioxidant, anti-inflammatory, and intestinal barrier-protective activities of its individual components, the astaxanthin–turmeric extract–honeysuckle extract combination (ATH) was investigated for its protective effects in a mouse model of dextran sulfate sodium (DSS)-induced colitis. Thirty-two male C57BL/6J mice were randomly allocated to four cohorts: Control, DSS, DSS + ATH, and ATH. ATH intervention mitigated the DSS-associated decline in body mass, lowered clinical disease scores, attenuated DSS-induced colon shortening, and ameliorated mucosal lesions. In DSS-treated mice, ATH attenuated goblet-cell loss, reduced intestinal permeability, and restored ZO-1 and occludin expression. ATH treatment also reduced DSS-induced increases in colonic IL-1β, IL-6, TNF-α, IL-18, and malondialdehyde levels while improving superoxide dismutase activity and glutathione content. At the signaling level, ATH reduced phosphorylated NF-κB and NLRP3 immunoreactivity and decreased N-GSDMD accumulation within E-cadherin-positive epithelial regions. Western blotting further showed reduced NLRP3 expression, caspase-1 cleavage, and N-GSDMD formation, although the p-NF-κB/NF-κB ratio was not significantly changed. Collectively, ATH alleviated DSS-induced colitis by improving mucosal barrier and redox–inflammatory homeostasis while attenuating the NLRP3/caspase-1/GSDMD pyroptotic cascade. These findings support the further development of ATH as a multi-component natural intervention for intestinal inflammatory injury.

## 1. Introduction

Inflammatory bowel disease (IBD) encompasses ulcerative colitis and Crohn’s disease and typically follows a protracted course characterized by alternating remission and relapse, with its worldwide disease burden continuing to expand. In 2021, approximately 3.83 million people worldwide were living with IBD, with nearly 375,000 new cases and more than 42,000 related deaths [1]. Ulcerative colitis involves chronic mucosal inflammation and epithelial disruption in the colon, resulting in relapsing diarrhea, hematochezia, abdominal discomfort, and reduced well-being [2]. Although current therapies can induce or maintain remission in some patients, their efficacy is variable and may be limited by relapse, adverse effects, or loss of response, underscoring the need to develop safe, multi-target interventions that restore mucosal homeostasis [2,3].

Disruption of the epithelial barrier is an early event that contributes substantially to the onset and persistence of colonic inflammation [4]. The mucus layer secreted by goblet cells and the tight junction complexes connecting adjacent epithelial cells together restrict the passage of luminal antigens and microbial products into the lamina propria [5,6]. Loss of this spatial separation exposes the mucosa to continuous inflammatory stimulation [4]. The resulting inflammatory response further promotes epithelial cell death, thereby aggravating barrier permeability and tissue damage [4]. Pyroptosis is an inflammatory form of regulated cell death that may connect inflammasome activation with the deterioration of epithelial integrity [7,8]. In the canonical pathway, nuclear factor kappa B (NF-κB) signaling provides the priming signal by inducing the expression of NLR family pyrin domain-containing 3 (NLRP3) and the synthesis of pro-inflammatory cytokine precursors [9]. Subsequent inflammasome formation activates caspase-1, which cleaves gasdermin D and releases its pore-forming N-terminal fragment, N-GSDMD [10,11]. Excessive N-GSDMD accumulation in intestinal epithelial cells can promote the release of inflammatory mediators, and this change is also related to goblet-cell loss, epithelial damage, and further development of colitis; therefore, reducing abnormal activation of the NF-κB/NLRP3/caspase-1/GSDMD pathway, while maintaining the epithelial barrier, may help lessen intestinal inflammation and mucosal injury [8,12].

Food-derived active components and plant extracts have been increasingly studied as possible treatments for colitis, because they may act on oxidative stress, inflammatory reactions, and epithelial barrier injury at the same time [13,14]. Astaxanthin is one of these compounds, and it is a xanthophyll carotenoid with strong antioxidant activity and a regulatory effect on inflammatory signaling [15,16]. Natural astaxanthin is mainly obtained from the green microalga *Haematococcus pluvialis* and the red yeast *Phaffia rhodozyma*; compared with synthetic astaxanthin, it comes from renewable biological sources, and some natural preparations have shown better tissue accumulation or higher antioxidant activity [17,18]. In dextran sulfate sodium (DSS)-induced colitis, astaxanthin reduced disease activity, oxidative damage, inflammatory cytokine production, and goblet cell depletion while suppressing mucosal NF-κB activation [19,20]. Astaxanthin-based delivery systems have also been shown to restore ZO-1 and occludin expression and improve intestinal barrier integrity. Curcumin, the principal active compound in turmeric, ameliorated DSS-evoked colonic injury through suppression of the NF-κB/NLRP3 inflammatory axis [21,22]. In parallel, *Lonicera japonica*-derived polysaccharides and chlorogenic acid have shown protective effects against DSS-induced mucosal injury, inflammatory cytokine production, and intestinal ecological disturbance [23,24]. These findings provide a pharmacological rationale for integrating astaxanthin, turmeric extract, and honeysuckle extract into a multi-component formulation capable of targeting the closely coupled redox, inflammatory, and barrier-related abnormalities of colitis.

However, whether a formulation containing astaxanthin, turmeric extract, and honeysuckle extract can concurrently modulate the closely interconnected epithelial barrier dysfunction, redox–inflammatory imbalance, and pyroptotic signaling associated with DSS-induced colitis remains unclear. Rather than addressing the individual efficacy or synergy of its constituents, the present study evaluated ATH at the formulation level and examined whether its protective phenotype was accompanied by coordinated changes in barrier function and NLRP3/caspase-1/GSDMD-associated epithelial pyroptosis. Accordingly, we assessed whether an astaxanthin–turmeric extract–honeysuckle extract formulation (ATH) could ameliorate DSS-evoked colitis in mice and explored the molecular events potentially contributing to its action. Disease activity, colonic histopathology, goblet cell preservation, intestinal permeability, and tight-junction protein expression were examined together with colonic inflammatory and redox indices. Furthermore, the activation of NF-κB, NLRP3, caspase-1, and GSDMD, as well as the epithelial distribution of N-GSDMD, was assessed to determine whether attenuation of epithelial pyroptosis contributes to ATH-mediated protection. Our working hypothesis was that ATH protects against DSS colitis through mucosal barrier fortification, restoration of redox–inflammatory balance, and suppression of NLRP3/caspase-1/GSDMD-driven pyroptosis.

## 2. Results

### 2.1. ATH Alleviated DSS-Induced Colitis Severity

**Figure 1:**
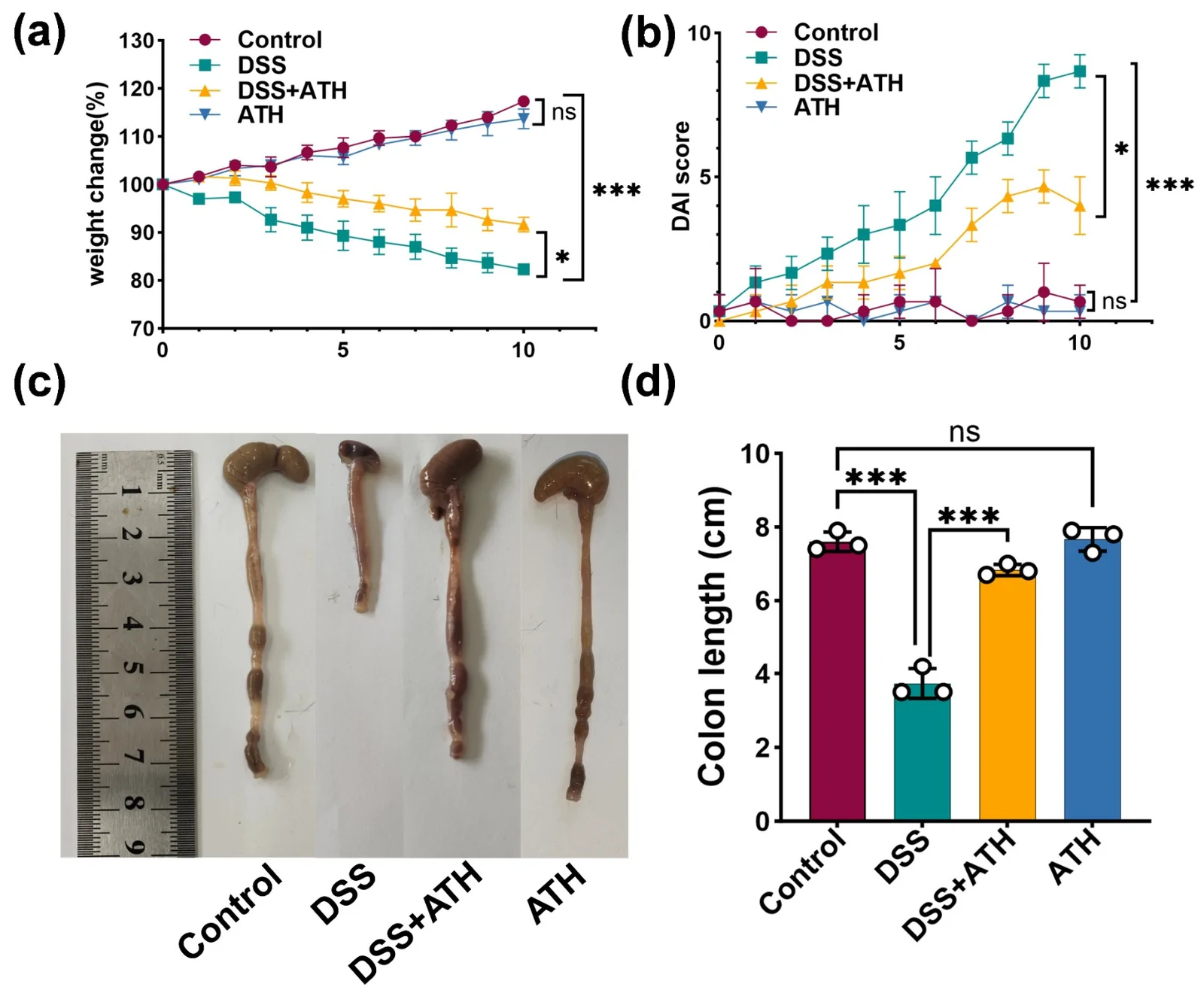
ATH attenuates disease severity in mice with DSS-induced colitis. (**a**) Changes in body weight. (**b**) Daily disease activity index (DAI) scores of mice in each group. (**c**) Representative gross images of colons collected from the indicated groups. (**d**) Quantification of colon length. ATH, astaxanthin–turmeric extract–honeysuckle extract combination; DSS, dextran sulfate sodium. Data are presented as mean ± SD (*n* = 8 mice per group for a and b; *n* = 3 mice per group for d). * *p* < 0.05 and *** *p* < 0.001 between the indicated groups; ns, not significant.

Body mass trajectories, disease activity index (DAI) scores, and colonic length were examined to assess the efficacy of ATH. Body weight continued to increase in the Control and ATH-only groups, while mice exposed to DSS showed a gradual decrease during the experiment; ATH administration reduced this DSS-related weight loss (Figure 1a). A similar change was found in the disease activity index, as DAI scores rose progressively after DSS treatment, showing that the clinical signs of colitis became more severe, whereas the increase was significantly smaller in the DSS + ATH group (Figure 1b). DSS treatment also caused an obvious reduction in colon length; this shortening was partly improved by ATH, as shown by the representative colon images and the quantitative results (Figure 1c,d). No significant differences in body weight, DAI, or colon length were detected between the ATH-only and Control groups. These results indicate that ATH reduced the clinical severity of DSS-induced colitis and did not noticeably affect these parameters in healthy mice. Serum biochemical indices were also examined to preliminarily assess systemic tolerability. DSS significantly increased alanine aminotransferase (ALT), aspartate aminotransferase (AST), and blood urea nitrogen (BUN) levels, whereas these elevations were attenuated by ATH treatment. No significant differences in these parameters were observed between the ATH-only and Control groups (Figure S1).

### 2.2. ATH Ameliorated Colonic Mucosal Injury, Preserved Goblet Cells, and Reduced Intestinal Permeability

**Figure 2:**
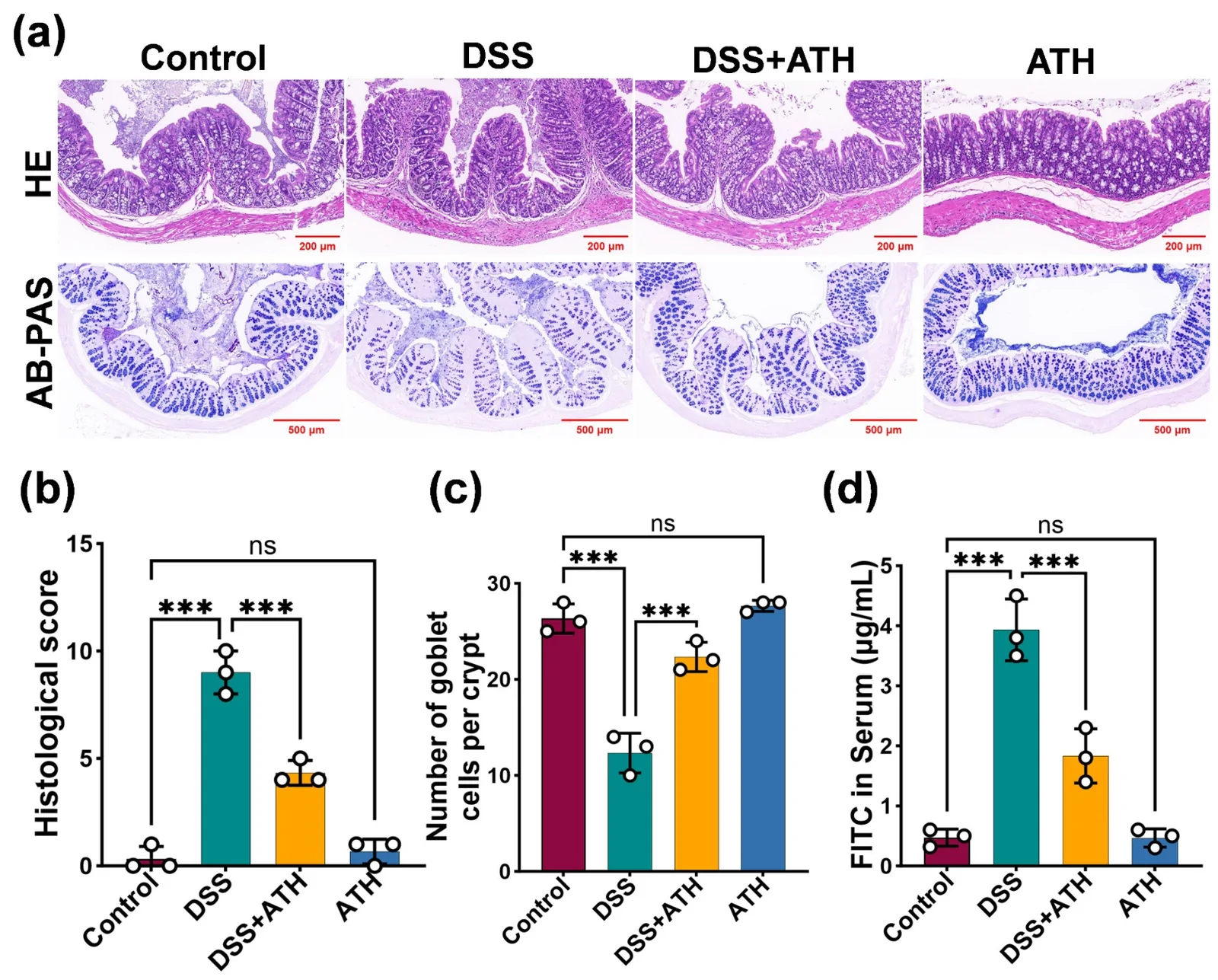
ATH attenuates DSS-induced colonic histopathological injury. (**a**) Representative images of hematoxylin and eosin (H&E) and Alcian blue–periodic acid–Schiff (AB-PAS) staining of colon tissues from the indicated groups. Scale bars = 200 μm for H&E staining and 500 μm for AB-PAS staining. (**b**) Quantification of colonic histological injury scores. (**c**) Quantification of AB-PAS-positive goblet cells. (**d**) Serum fluorescein isothiocyanate (FITC)-dextran concentration, used as an indicator of intestinal permeability. Data are presented as mean ± SD (*n* = 3 mice per group). *** *p* < 0.001 between the indicated groups; ns, not significant.

Histological examination was used to assess whether ATH affected the colonic mucosal injury caused by DSS. In the Control and ATH-only groups, the mucosal structure was generally complete, the crypts were arranged in a regular manner, and the epithelial layer was well preserved; however, DSS treatment caused clear tissue damage, including distorted or missing crypts, interruption of the epithelial lining, and obvious inflammatory cell infiltration. These changes were less severe in the DSS + ATH group (Figure 2a), and the histological injury score showed the same trend, as DSS increased the score while ATH treatment significantly lowered it (Figure 2b).

Alcian blue–periodic acid–Schiff (AB-PAS) staining also showed that DSS reduced the number of mucin-containing goblet cells in the colonic crypts; the quantitative results indicated that ATH partly reduced this loss (Figure 2a,c). Serum FITC-dextran was then measured to evaluate intestinal permeability and DSS caused a clear increase in serum FITC-dextran compared with the Control group, whereas ATH treatment lowered its entry into the blood circulation (Figure 2d). No significant differences in histological score, goblet-cell number, or serum FITC-dextran level were found between the Control and ATH-only groups; these results indicate that ATH reduced DSS-related mucosal injury, limited goblet-cell loss, and improved intestinal barrier function.

### 2.3. ATH Restored Colonic Epithelial Tight-Junction Integrity in DSS-Treated Mice

**Figure 3:**
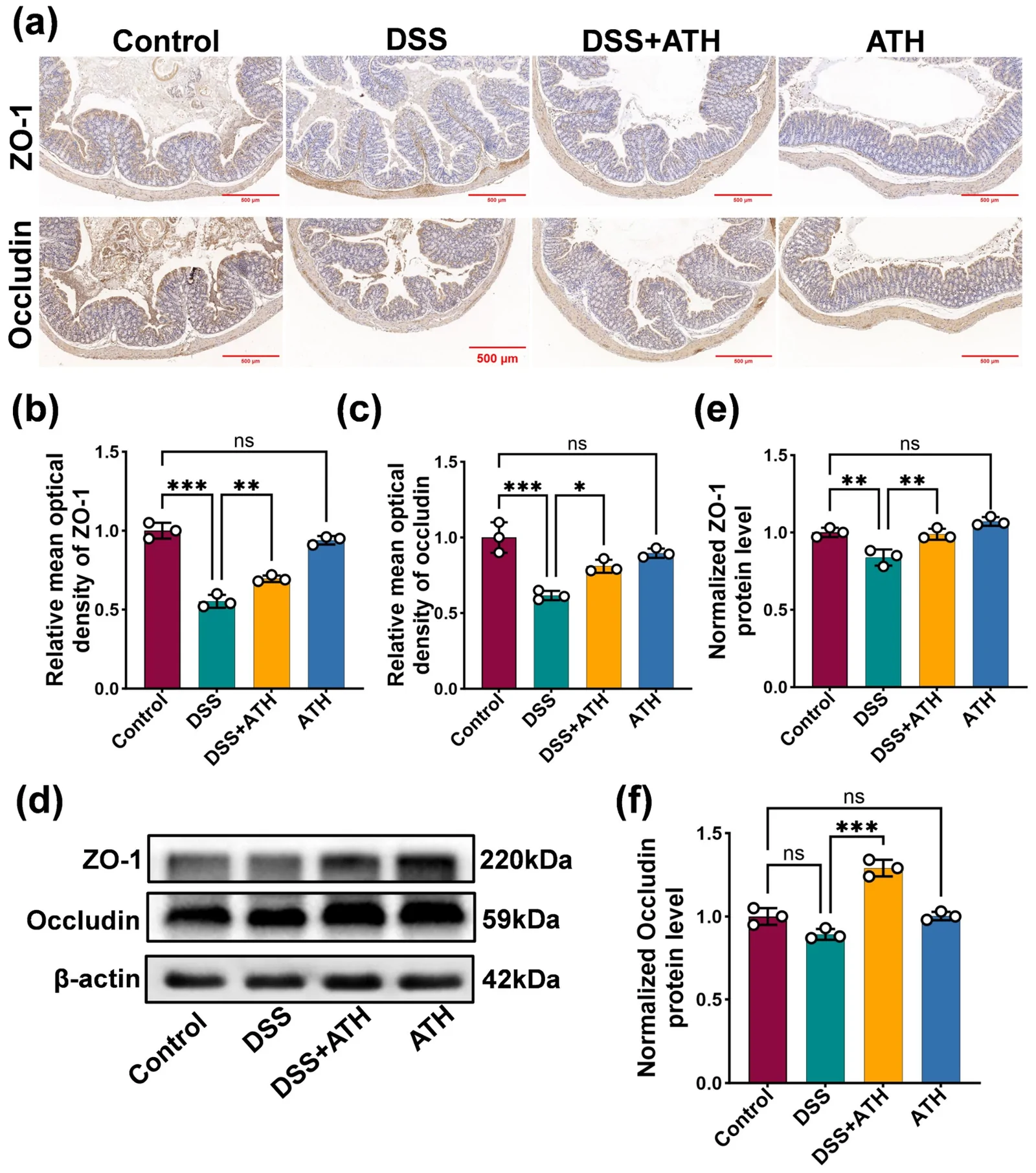
ATH preserves colonic tight junction protein expression in DSS-treated mice. (**a**) Representative immunohistochemical images of ZO-1 and occludin in colon tissues. Scale bars = 500 μm. (**b**,**c**) Quantitative analysis of the mean staining density of ZO-1 and occludin, respectively. Brown staining indicates positive ZO-1 or Occludin expression, and blue staining represents hematoxylin-counterstained nuclei. (**d**) Representative immunoblots of ZO-1 and occludin in colon tissues. (**e**,**f**) Densitometric quantification of ZO-1 and occludin protein levels. Data are presented as mean ± SD (*n* = 3 mice per group). * *p* < 0.05, ** *p* < 0.01, and *** *p* < 0.001 between the indicated groups; ns, not significant.

Following the ATH-mediated reduction in gut permeability, colonic ZO-1 and occludin were examined by immunohistochemistry. Both proteins were readily detected in the epithelial layer of Control mice but showed markedly diminished staining after DSS exposure. ATH administration partially restored ZO-1 and occludin immunoreactivity in DSS-treated mice (Figure 3a). Quantification corroborated the immunohistochemical observations: DSS diminished colonic ZO-1 and occludin staining, whereas ATH significantly counteracted these reductions (Figure 3b,c).

Western blot analysis further showed that DSS exposure significantly reduced ZO-1 protein abundance relative to the Control group, whereas ATH administration restored ZO-1 expression (Figure 3d,e). Although DSS elicited only a nonsignificant decline in total occludin, ATH treatment significantly increased its abundance relative to DSS alone (Figure 3d,f). These data support a role for ATH in maintaining epithelial junctional architecture and alleviating DSS-associated barrier dysfunction.

### 2.4. ATH Suppressed Colonic Inflammatory Responses and Oxidative Stress

**Figure 4:**
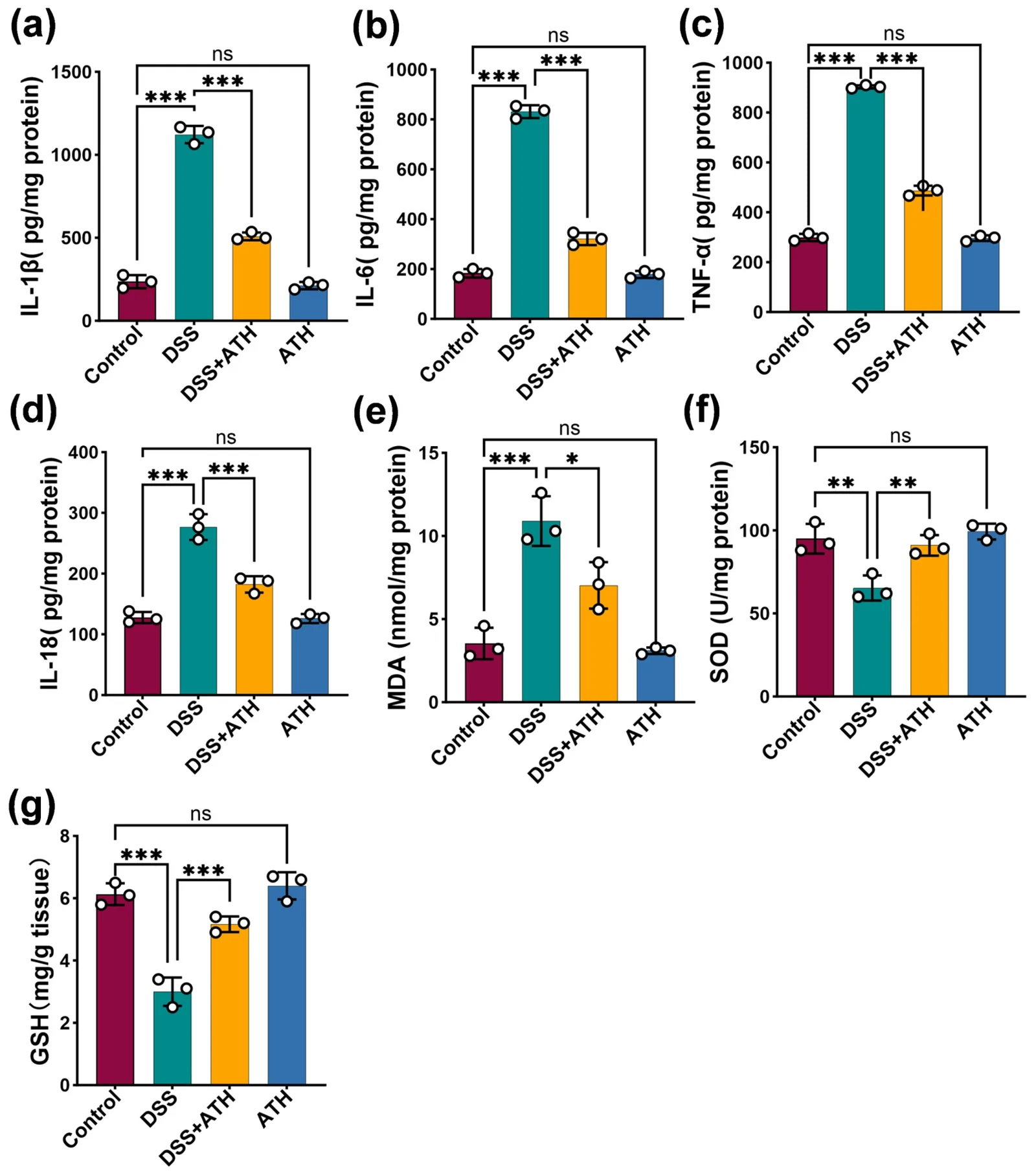
ATH suppresses DSS-induced colonic inflammation and oxidative stress in mice. (**a**–**d**) IL-1β, IL-6, TNF-α, and IL-18 levels in colon tissues from the indicated groups. (**e**) MDA level. (**f**) SOD activity. (**g**) GSH content. Data are presented as mean ± SD (*n* = 3 mice per group). * *p* < 0.05, ** *p* < 0.01, and *** *p* < 0.001 between the indicated groups; ns, not significant.

To assess the local inflammatory response, the levels of IL-1β, IL-6, TNF-α, and IL-18 were measured in colonic tissues. All four cytokines were elevated after DSS administration compared with the Control group. ATH treatment lowered these increases, although the cytokine levels in the DSS + ATH group remained above those of untreated mice (Figure 4a–d).

Changes in colonic oxidative status were further evaluated using malondialdehyde (MDA), superoxide dismutase (SOD), and glutathione (GSH) as representative biomarkers. DSS increased MDA accumulation, whereas this effect was partly counteracted by ATH treatment (Figure 4e). By contrast, SOD activity and GSH content declined following DSS challenge and increased toward Control levels after ATH administration (Figure 4f,g). The ATH-only group showed no significant changes in any of these parameters relative to the Control group. Taken together, these findings suggest that ATH attenuated the inflammatory and oxidative disturbances induced by DSS, as reflected by lower colonic cytokine and MDA levels and improved endogenous antioxidant capacity.

### 2.5. ATH Suppressed Activation of the NF-κB/NLRP3/Caspase-1/N-GSDMD Pyroptotic Cascade in Colonic Tissues

**Figure 5:**
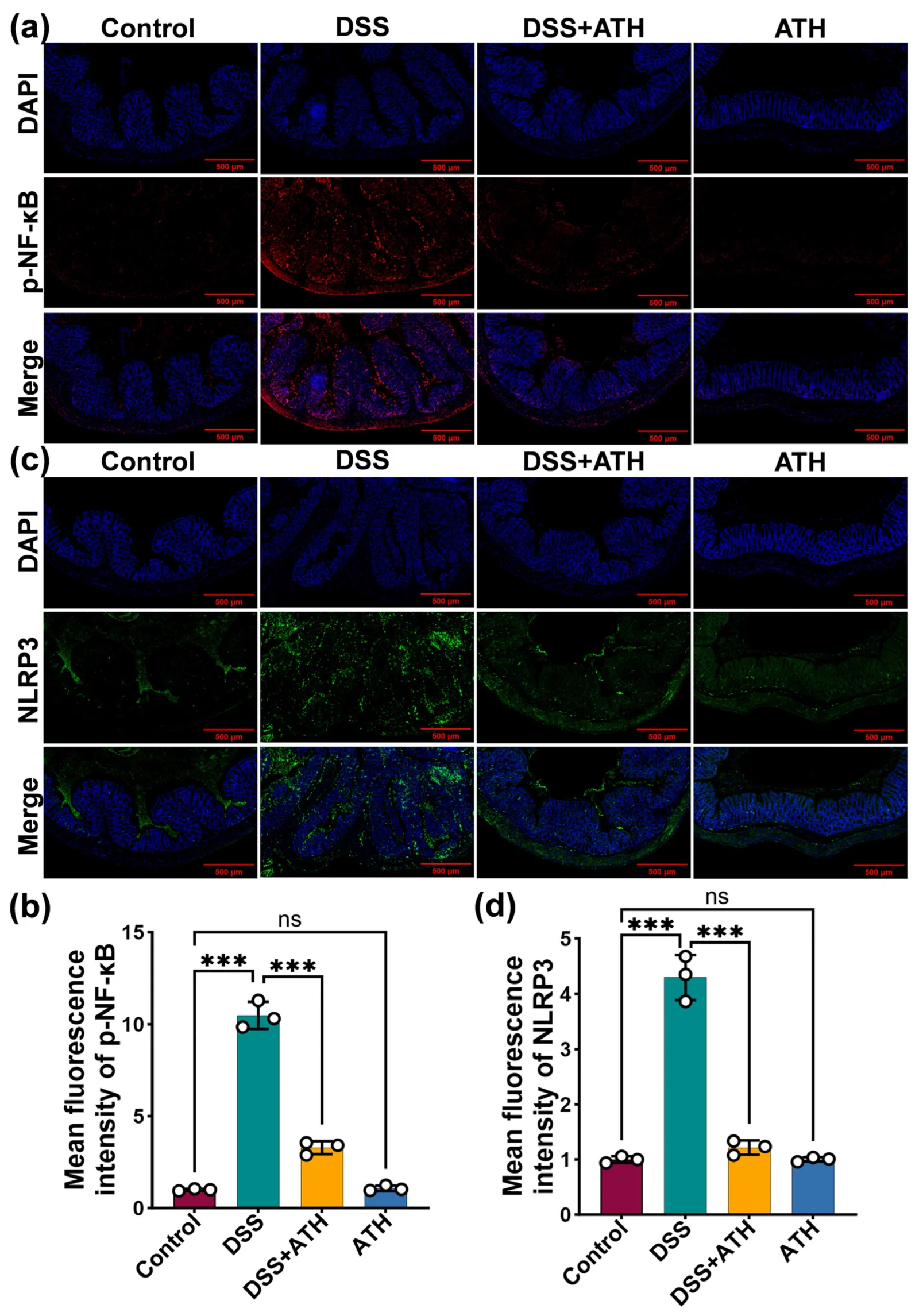
ATH attenuates DSS-induced upregulation of phosphorylated NF-κB and NLRP3 in mouse colon tissues. (**a**) Representative immunofluorescence images of p-NF-κB in colon tissues from the indicated groups. p-NF-κB is shown in red, and nuclei were counterstained with DAPI in blue. (**b**) Quantification of the mean fluorescence intensity of p-NF-κB. (**c**) Representative immunofluorescence images of NLRP3 in colon tissues. NLRP3 is shown in green, and nuclei were counterstained with DAPI in blue. (**d**) Quantification of the mean fluorescence intensity of NLRP3. Scale bars = 500 μm. Data are presented as mean ± SD (*n* = 3 mice per group). *** *p* < 0.001 between the indicated groups; ns, not significant.

The contribution of inflammatory and pyroptotic signaling to ATH-mediated protection was examined by immunofluorescent detection of p-NF-κB and NLRP3 in colon sections. DSS exposure markedly increased p-NF-κB immunoreactivity in colon tissues, whereas ATH administration substantially reduced this increase (Figure 5a,b). Similarly, NLRP3 fluorescence intensity was markedly elevated following DSS exposure and was restored toward control levels by ATH treatment (Figure 5c,d). Neither p-NF-κB nor NLRP3 fluorescence intensity differed significantly between the ATH and Control groups.

**Figure 6:**
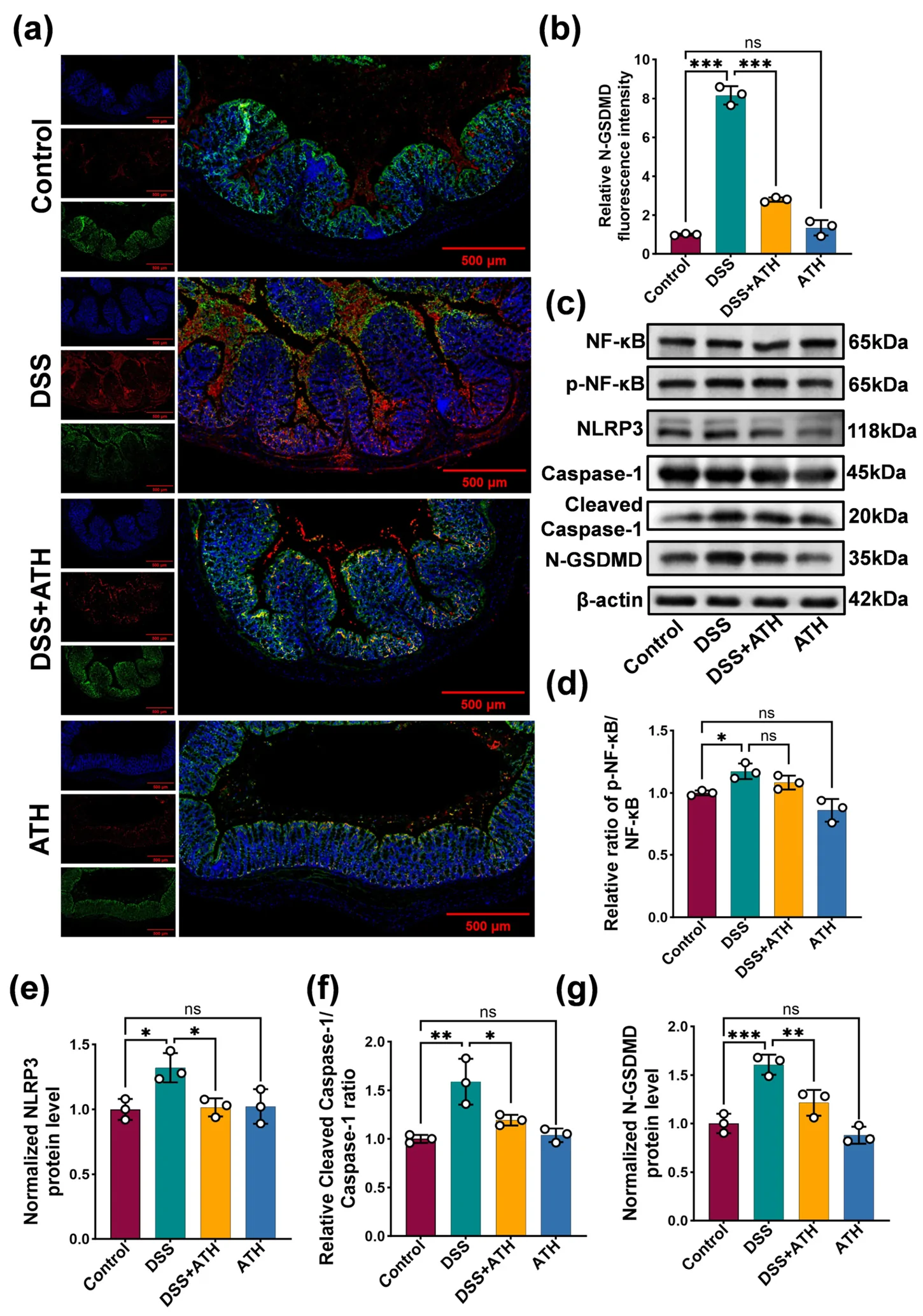
ATH attenuates activation of the NF-κB/NLRP3/caspase-1/GSDMD pyroptosis pathway in DSS-induced colitis. (**a**) Representative double-immunofluorescence images showing the distribution of N-GSDMD and E-cadherin in colon tissues from the indicated groups. N-GSDMD is shown in red, E-cadherin in green, and nuclei were counterstained with DAPI in blue. Scale bars = 500 μm. (**b**) Quantification of the relative N-GSDMD fluorescence intensity within E-cadherin-positive epithelial regions. (**c**) Representative immunoblots of NF-κB, p-NF-κB, NLRP3, caspase-1, cleaved caspase-1, and N-GSDMD in colon tissues. (**d**) Densitometric quantification of the p-NF-κB/NF-κB ratio. (**e**) Quantification of NLRP3 protein expression. (**f**) Quantification of the cleaved caspase-1/caspase-1 ratio. (**g**) Quantification of N-GSDMD protein expression. Data are presented as mean ± SD (*n* = 3 mice per group). * *p* < 0.05, ** *p* < 0.01, and *** *p* < 0.001 between the indicated groups; ns, not significant.

To determine whether epithelial pyroptosis was involved, double-immunofluorescence staining of N-GSDMD and E-cadherin was subsequently performed. DSS treatment induced a pronounced accumulation of N-GSDMD signals within E-cadherin-positive colonic epithelial regions, whereas this increase was visibly attenuated by ATH administration (Figure 6a). Quantification of epithelial N-GSDMD immunofluorescence revealed a marked elevation following DSS challenge, whereas ATH intervention substantially blunted this response (Figure 6b). The signal intensity in mice receiving ATH alone remained comparable to that in untreated controls.

Western blot analysis was further used to evaluate key components of the NF-κB/NLRP3/caspase-1/N-GSDMD cascade. DSS exposure significantly increased the p-NF-κB/NF-κB ratio. Although a downward trend was observed following ATH administration, the difference between the DSS and DSS + ATH groups did not reach statistical significance (Figure 6c,d). By contrast, DSS treatment significantly increased NLRP3 protein expression, the cleaved caspase-1/caspase-1 ratio, and N-GSDMD protein abundance relative to the Control group. ATH administration significantly reduced NLRP3 expression, caspase-1 cleavage, and N-GSDMD accumulation compared with DSS treatment alone (Figure 6c,e–g). These protein indices did not differ significantly between the ATH and Control groups. Collectively, the immunofluorescence and immunoblotting results indicate that ATH attenuated DSS-induced activation of the NLRP3/caspase-1/N-GSDMD pyroptotic cascade. For NF-κB, ATH significantly reduced p-NF-κB immunoreactivity by immunofluorescence, whereas the p-NF-κB/NF-κB ratio showed only a nonsignificant downward trend by Western blotting.

## 3. Discussion

The present study identifies a coordinated pattern of ATH-associated protection against DSS-induced colitis, characterized by preservation of epithelial barrier homeostasis, improvement of redox–inflammatory status, and attenuation of inflammasome-associated pyroptotic signaling. The protective phenotype was not confined to an improvement in clinical manifestations but was accompanied by consistent changes across the mucosal barrier, redox status, inflammatory response, and pyroptotic machinery. In particular, restoration of goblet cells and tight-junction proteins occurred in parallel with reduced intestinal permeability, lower inflammatory cytokine production, and suppression of the NLRP3/caspase-1/N-GSDMD cascade. These results are consistent with ATH weakening the reciprocal interaction between epithelial damage, increased permeability, local inflammation, and pyroptotic cell loss. N-GSDMD signals were mainly detected within E-cadherin-positive areas, indicating that pyroptotic activity occurred in the epithelial compartment. This spatial overlap also links epithelial pyroptosis with disruption of the mucosal barrier.

Preservation of epithelial integrity may therefore represent an important component of the protection afforded by ATH. The mucus layer and intercellular junctions defend the mucosa through different but complementary mechanisms. Mucins released by goblet cells separate luminal material from the epithelial surface, whereas ZO-1 and occludin help control paracellular passage [5,25]. DSS compromised both defenses, thereby facilitating the movement of microbial constituents and dietary antigens into the underlying tissue and prolonging mucosal inflammation [4,6]. In mice treated with ATH, the reduction in goblet cells was less obvious, and the expression of ZO-1 and occludin was maintained to a greater extent; serum FITC–dextran levels were also lower than those in the DSS group. These results indicate that ATH improved the tissue structure of the intestinal barrier and also reduced the increase in intestinal permeability. This explanation is in agreement with previous studies, which reported that astaxanthin reduced goblet-cell loss and helped maintain epithelial structure in DSS-induced colitis [19,20]. Preparations containing astaxanthin have also been shown to increase the levels of MUC2, ZO-1, and occludin; similar effects on intestinal barrier protection have been reported for curcumin [22] and several components obtained from *Lonicera japonica* [26]. In light of these reports, our data suggest that ATH helps retain the physical separation between the intestinal lumen and lamina propria, thereby reducing persistent exposure of mucosal immune cells to pro-inflammatory luminal stimuli.

Oxidative stress and inflammatory signaling form another interconnected component of DSS-induced mucosal injury [27]. Excessive lipid peroxidation can impair epithelial membranes and tight-junction organization, whereas depletion of endogenous antioxidants increases the responsiveness of epithelial and immune cells to inflammatory stimuli [27]. The reduction in MDA and recovery of SOD activity and GSH content after ATH administration therefore indicate a shift toward a less oxidative colonic microenvironment. This redox improvement was accompanied by lower IL-1β, IL-6, and TNF-α levels and reduced mucosal p-NF-κB immunoreactivity. However, the p-NF-κB/NF-κB ratio measured by Western blotting showed only a nonsignificant downward trend after ATH treatment, indicating that the NF-κB-related effect was more evident in the section-based immunofluorescence readout than in the whole-tissue phosphorylation ratio. NF-κB-dependent transcription promotes the expression of NLRP3 and pro-IL-1β, whereas oxidative disturbances and related danger signals facilitate subsequent inflammasome activation [9,27]. ATH may therefore influence both the priming and activation stages of the inflammatory cascade. Earlier studies provide some support for this explanation, as astaxanthin was reported to reduce NF-κB nuclear translocation and inflammatory cytokine release in DSS-induced colitis [19]; curcumin was also found to decrease NLRP3 inflammasome activation by reducing intracellular ROS levels, potassium efflux, and cathepsin B release [21]. These findings indicate that the antioxidant and anti-inflammatory effects of ATH may be related to each other, rather than representing two separate pharmacological actions.

Another result was that ATH reduced the activity of the NLRP3/caspase-1/GSDMD pathway. After the inflammasome is formed, caspase-1 becomes activated and cleaves GSDMD, which releases the N-terminal fragment that can form pores in the cell membrane; these pores allow inflammatory substances to leave the cell and can finally cause pyroptotic cell death [10,11]. When this process is overactivated in intestinal epithelial cells, the continuity of the epithelial layer may be damaged, and local mucosal inflammation may become more severe [8,28]. In this study, ATH treatment lowered NLRP3 expression, caspase-1 cleavage, and N-GSDMD accumulation, a pattern consistent with attenuation of several components of the canonical pyroptosis-related signaling cascade. Consistent with these molecular changes, ATH also significantly reduced the DSS-induced elevation of colonic IL-18, providing an additional downstream readout associated with attenuation of inflammasome/caspase-1 signaling. The N-GSDMD fluorescence signal was also decreased in E-cadherin-positive areas; this result suggests that the change occurred, at least partly, in the epithelial region involved in maintaining the intestinal barrier. Similar results have been reported in experimental colitis, where reducing caspase-1/GSDMD-related epithelial pyroptosis was accompanied by better tight-junction structure and lower intestinal permeability [8,29]. Conversely, GSDMD activation has been linked to IL-18 release and worsening colonic inflammation, whereas genetic deletion of GSDMD, either alone or together with GSDME, lessens DSS-associated tissue damage and inflammatory responses [30]. These findings support epithelial pyroptosis-related signaling as a potential link between inflammatory activation and barrier dysfunction, although its causal contribution to the protective effect of ATH remains to be established. Nevertheless, inflammasome activity is not uniformly detrimental, because basal signaling contributes to epithelial turnover and mucosal defense [31,32]. ATH should therefore be viewed as tempering pathological inflammasome overactivation during DSS challenge rather than abolishing NLRP3 signaling altogether.

The formulation-level activity observed for ATH may reflect contributions from its three components, although the present study does not identify the individual chemical constituent responsible for these effects. Astaxanthin readily partitions into lipid bilayers, where it can limit oxidative injury; in DSS models, it has also been associated with weaker NF-κB signaling, lower cytokine production, and reduced goblet-cell depletion [16,19]. Turmeric-derived curcuminoids act on several inflammatory pathways and may curb NLRP3 inflammasome activity as well as caspase-1-mediated maturation of IL-1β [21,22]. Phenolic constituents of honeysuckle extract have likewise been associated with reduced colonic oxidative stress and inflammation and improved mucosal barrier function [23,26]. The concurrent changes observed in the present study are therefore unlikely to reflect modulation of a single target. Instead, ATH may act at several linked sites, including oxidative imbalance, inflammatory signaling, epithelial disruption, and pyroptotic injury. Such a mode of action is pertinent to DSS colitis, in which direct epithelial damage occurs alongside innate immune activation and inadequate barrier repair.

Several limitations should be considered. The present study was designed as a formulation-level evaluation of ATH rather than a component-resolved comparison of its individual constituents. First, the present design did not include the individual ingredients or pairwise combinations. Consequently, the relative contribution of astaxanthin, turmeric extract, and honeysuckle extract, as well as potential additive or synergistic interactions, remains to be determined [33]. Component-resolved factorial experiments, together with comprehensive chromatographic fingerprinting and batch-specific quantitative characterization of the major constituent classes, will be required to clarify the material basis of ATH activity and to determine whether individual chemical constituents can be linked to the observed biological effects. In addition, the 150:200:200 mg/kg formulation ratio was not formally optimized, and systematic dose–response and mixture-design studies will be required to determine whether alternative component ratios modify the efficacy or tolerability of ATH. Second, concomitant changes in NLRP3, cleaved caspase-1, and N-GSDMD implicate pyroptosis but cannot demonstrate its causal role. This mechanism requires further confirmation using pathway-selective inhibitors, GSDMD-null models, intestinal organoids, or primary intestinal epithelial cells. Future studies using intestinal epithelial cells, organoids, or primary epithelial cultures combined with extracellular LDH release and membrane-integrity assays would provide more direct evidence of pyroptotic cell death. Third, the present study did not include metabolomic profiling. Metabolomic analysis of colonic tissue, serum, or fecal samples, followed by targeted validation of candidate metabolites, may help identify metabolic responses associated with ATH intervention and provide additional mechanistic hypotheses. Integration of such metabolic information with epithelial-cell or organoid experiments would further clarify whether specific metabolic changes participate in the barrier-protective and inflammasome-associated responses observed in vivo. Fourth, acute DSS challenge chiefly models epithelial injury and innate immune activation and therefore does not fully mirror the relapsing course or immune complexity of human inflammatory bowel disease [34,35]. Validation in chronic or recurrent colitis models would therefore strengthen the translational relevance of the findings. Collectively, our findings indicate that ATH mitigates DSS-evoked colonic injury, with this protective phenotype accompanied by preservation of mucosal integrity, improvement of redox–inflammatory homeostasis, and attenuation of epithelial NLRP3/caspase-1/GSDMD-associated signaling. Accordingly, the contribution of the present study lies in demonstrating, at the formulation level, a coordinated pattern of epithelial barrier preservation, improved redox–inflammatory homeostasis, and attenuated epithelial pyroptosis-related signaling. Component-resolved combination studies, mechanistic validation in epithelial-cell models, and metabolomic profiling therefore represent important priorities for subsequent investigation.

## 4. Materials and Methods

### 4.1. Reagents

Astaxanthin (95%) was provided by Jilin Wanfang Dongxun Astaxanthin Industrial Technology Development Co., Ltd. (Baishan, China). Turmeric (*Curcuma longa* L.) extract and honeysuckle (*Lonicera japonica* Thunb.) extract (extraction ratio, 10:1) were purchased from Shanghai Rhawn Chemical Technology Co., Ltd. (Shanghai, China). The chemical characteristics and contents of the major curcuminoids in the turmeric extract and the major marker constituents in the honeysuckle extract are presented in Tables S1 and S2, respectively.

### 4.2. Experimental Animals and Study Design

Thirty-two male C57BL/6J mice (4–6 weeks, 18–22 g) were supplied by SPF (Beijing) Biotechnology Co., Ltd. (Beijing, China). They were housed at the Experimental Animal Center of Jilin University under barrier conditions (22 ± 2 °C; 12 h light/dark cycle), with food and water provided ad libitum. All procedures received approval from the Animal Ethics Committee of Jilin University (Approval No. 2026-606). Eight mice were allocated to each group on the basis of earlier acute DSS studies and the 3Rs framework, with DAI defined a priori as the primary endpoint [34,36]. DSS was purchased from Shanghai Yuanye Bio-Technology Co., Ltd. (Shanghai, China).

After a 7-day acclimatization period, the mice were randomly allocated to four groups (n = 8 per group): Control, DSS, DSS + ATH, and ATH. Mice in the Control group received normal drinking water and an equivalent volume of corn oil by oral gavage once daily. Mice in the DSS group were given 2.5% (*w*/*v*) DSS in drinking water ad libitum on days 1–7 and an equivalent volume of corn oil by oral gavage once daily on days 1–10. Mice in the DSS + ATH group were given 2.5% DSS in drinking water on days 1–7 and orally administered the ATH once daily on days 1–10. Mice in the ATH group received normal drinking water and ATH by oral gavage once daily on days 1–10. The DSS solution was freshly prepared and replaced every 2 days [35,37].

ATH provided astaxanthin, turmeric extract, and honeysuckle extract at doses of 150, 200, and 200 mg/kg body weight, respectively. The astaxanthin dose was derived from a dietary inclusion rate of 0.1% (*w*/*w*). Assuming that a 20 g mouse consumed approximately 3 g of feed per day, the corresponding daily intake was estimated at 3 mg per animal, or 150 mg/kg body weight. Turmeric and honeysuckle extract doses were chosen with reference to those used in earlier in vivo studies [23,38]. The quantitative composition of the ATH formulation is provided in Table S3. These component doses were combined to establish an initial ATH formulation for the present proof-of-concept study. The resulting 150:200:200 mg/kg ratio was not derived from a preliminary combination-ratio optimization experiment and should therefore not be interpreted as an experimentally optimized dose ratio. All eight mice per group were included in the longitudinal clinical assessments, including body-weight change and DAI. A predetermined subset of three mice per group was used for terminal gross and tissue-based analyses, including colon-length measurement, histological assessment, biochemical assays, immunohistochemistry, immunofluorescence, and Western blotting. Animals included in this subset were designated before the corresponding downstream assay results were available, and selection was not based on disease severity or subsequent experimental outcomes.

### 4.3. Clinical Monitoring and Disease Activity Index

Body weight and general clinical condition were checked at nearly the same time every day, and the daily examination included stool form, occult or visible fecal blood, spontaneous activity, grooming, and food and water intake. Changes in body weight were calculated as percentages of the value recorded on day 0. Disease activity was evaluated using a combined disease activity index based on body weight loss, stool consistency, and fecal bleeding; the total score ranged from 0 to 12 [35,37]. Weight loss was scored as 0 when no loss occurred, 1 for a reduction of 1–5%, 2 for >5–10%, 3 for >10–15%, and 4 for >15%. Normal formed stools were given a score of 0, loose stools were scored as 2, and watery diarrhea was scored as 4; fecal bleeding was scored as 0 when no blood was found, 2 when occult blood was detected, and 4 when visible rectal bleeding was present.

Each mouse was observed at least once every day, and additional checks were carried out when abnormal signs appeared. Humane endpoints were set before the experiment; these included loss of more than 20% of the initial body weight, continued inability to obtain food or water, marked reduction in activity, persistent heavy rectal bleeding, rectal prolapse, breathing difficulty, or a moribund state. Any mouse meeting one of these conditions was to be euthanized immediately, and the related information was to be recorded. Analgesic and anti-inflammatory drugs were not routinely given because they might affect the inflammatory measurements; animal handling was reduced as much as possible, and oral gavage and terminal procedures were completed by trained staff.

Neither animals nor individual measurements were removed only because they appeared to be statistical outliers. Exclusion was allowed only for reasons determined before the study, such as serious injury caused by gavage, accidental death unrelated to DSS treatment, incorrect administration, sample loss, or technical failure; no mouse reached a humane endpoint, showed an unexpected adverse event, or was excluded from the final dataset.

### 4.4. FITC-Dextran Permeability Assay and Sample Collection

After the last clinical examination on day 10, the predefined terminal-analysis subset (n = 3 mice per group) was used for the FITC-dextran permeability assay and subsequent sample collection. These mice were deprived of food for 4 h with free access to water. FITC-labeled dextran (4 kDa; Yeasen Biotechnology Co., Ltd., Shanghai, China) was prepared in sterile phosphate-buffered saline and administered by oral gavage at 600 mg/kg body weight. Four hours after administration, the mice were anesthetized with isoflurane, and blood samples were collected from the retro-orbital venous plexus [39]. Serum was separated by centrifugation at about 3000× *g* for 10 min and then stored at −80 °C until further detection. The fluorescence of 4 kDa FITC-dextran in serum was measured with a microplate reader, using excitation and emission wavelengths of about 485 and 528 nm, respectively; after blood collection, the mice were euthanized by carbon dioxide inhalation according to the approved animal protocol. For the predefined terminal-analysis subset, the whole colon was removed, the intestinal contents were gently cleaned away, and colon length was measured without stretching the tissue. Distal colon tissues from this subset were placed in 4% paraformaldehyde for histological examination and staining, while the remaining tissue samples were divided into several portions, rapidly frozen in liquid nitrogen, and stored at −80 °C for biochemical assays and Western blot analysis.

### 4.5. Histological and AB–PAS Staining

Distal colon tissues were fixed in paraformaldehyde, then dehydrated with graded ethanol, cleared in xylene, embedded in paraffin, and cut into 5 μm sections. The tissue sections were stained with hematoxylin and eosin following a routine procedure; images from all groups were collected under the same light-microscope settings. A researcher who did not know the group information evaluated the degree of colon injury, including inflammatory severity, depth of inflammatory cell infiltration, and crypt damage. Inflammatory severity and infiltration depth were scored from 0 to 3, while crypt damage was scored from 0 to 4; therefore, the total score ranged from 0 to 10, and a higher score represented more serious mucosal injury. For each mouse, at least three nonconsecutive sections taken at fixed intervals were examined, and five nonoverlapping mucosal areas were evaluated in each section; the average score of each mouse was regarded as one biological replicate.

Goblet cells and mucin distribution were visualized with an AB–PAS staining kit according to the supplier’s protocol. At least 10 well-oriented, longitudinally sectioned crypts were counted for each mouse across three nonconsecutive sections. Crypts lacking an intact base or a clearly visible luminal opening were omitted from the analysis. Goblet-cell abundance was reported as the mean number of AB–PAS-positive cells per crypt.

### 4.6. Immunohistochemistry

Dewaxed colon sections were subjected to citrate-based heat retrieval, followed by blockade of endogenous peroxidase and nonspecific binding with 3% H_2_O_2_ and 5% bovine serum albumin, respectively. After overnight incubation with anti-ZO-1 or anti-occludin antibodies at 4 °C, HRP-linked secondary antibodies were applied, and staining was quantified using ImageJ 1.54p (National Institutes of Health, Bethesda, MD, USA).

### 4.7. Measurement of Colonic Cytokines, Oxidative-Stress Indices, and Serum Biochemical Parameters

IL-1β, IL-6, TNF-α, and IL-18 concentrations in colonic homogenates were measured using ELISA kits from Jianglai Biotechnology Co., Ltd. (Shanghai, China). Colonic MDA, GSH, and SOD were assayed with kits from Nanjing Jiancheng Bioengineering Institute (Nanjing, China), according to the suppliers’ protocols. Serum ALT and AST activities and BUN levels were determined using commercial assay kits from Beijing Solarbio Science & Technology Co., Ltd. (Beijing, China), according to the manufacturer’s instructions.

### 4.8. Immunofluorescence and Double-Immunofluorescence Staining

Colon sections were deparaffinized, rehydrated, and subjected to antigen retrieval. After permeabilization with Triton X-100 and blocking with 5% bovine serum albumin, sections were incubated overnight at 4 °C with antibodies against phosphorylated NF-κB, NLRP3, N-GSDMD, or E-cadherin.

### 4.9. Western Blotting

Colon tissues were homogenized in ice-cold RIPA lysis buffer (Beijing Solarbio Science & Technology Co., Ltd., Beijing, China) supplemented with protease and phosphatase inhibitor cocktails. Lysates were centrifuged at 12,000× g for 15 min at 4 °C, and protein concentrations were measured using a bicinchoninic acid assay kit (Beijing Solarbio Science & Technology Co., Ltd., Beijing, China). Equal amounts of protein, approximately 20 μg per lane, were separated using 7.5–12.5% SDS-polyacrylamide gels selected according to the molecular weight of the target protein and transferred onto polyvinylidene difluoride membranes. Following transfer, membranes were sectioned according to the expected molecular-weight regions of the target proteins. Membranes were incubated overnight at 4 °C with primary antibodies (Table S4). Protein bands were visualized using an enhanced chemiluminescence reagent (Thermo Fisher Scientific, Waltham, MA, USA). β-actin was used as the loading control for protein-expression analyses, whereas p-NF-κB and cleaved caspase-1 were evaluated relative to total NF-κB and total caspase-1, respectively. Band intensities were quantified using ImageJ version 1.54p (National Institutes of Health, Bethesda, MD, USA).

### 4.10. Statistical Analysis

Statistical analyses were performed using GraphPad Prism 10.1.2. Data are presented as the mean ± SD, with the exact biological sample size reported in each figure legend. Longitudinal body weight and DAI data were analyzed using two-way repeated-measures analysis of variance, with group and time as factors, followed by Šídák’s multiple-comparison test when appropriate. Endpoint measurements involving the four groups were analyzed using one-way analysis of variance followed by Tukey’s multiple-comparison test. Because several secondary tissue-based analyses contained three biological replicates per group, formal normality testing was considered an auxiliary diagnostic rather than definitive evidence of distributional form. The use of parametric analysis was based on the prespecified analytical framework, the balanced design with equal group sizes, independence of biological replicates, and the absence of obvious extreme observations or marked distributional irregularities. Normality and variance homogeneity were additionally examined using the Shapiro–Wilk and Brown–Forsythe tests, respectively. When variance homogeneity was not satisfied, Welch’s analysis of variance followed by Dunnett’s T3 test was used. Data that did not meet the assumptions of parametric analysis were evaluated using the Kruskal–Wallis test followed by Dunn’s multiple-comparison test. No observation was removed solely to achieve statistical significance. A two-sided *p* value < 0.05 was considered statistically significant.

## 5. Conclusions

In conclusion, ATH alleviated DSS-induced colitis and was associated with improved mucosal barrier integrity and colonic redox–inflammatory homeostasis. These changes were accompanied by attenuation of NLRP3/caspase-1/GSDMD-associated pyroptotic signaling. The present findings provide formulation-level in vivo evidence supporting further investigation of ATH, while component-specific contributions, potential synergistic interactions, and causal mechanisms require additional validation.

## Data Availability

Data will be made available on request.

## Supplementary Materials

The following supporting information can be downloaded at: https://www.mdpi.com/article/10.3390/molecules31183303/s1, Figure S1. Effects of ATH on serum biochemical indices associated with hepatic and renal function in DSS-induced colitis; Table S1: Chemical characteristics and contents of major curcuminoids in the turmeric extract; Table S2: Chemical characteristics and contents of major marker constituents in the honeysuckle extract; Table S3: Composition and weight-based dosing of the ATH formulation used in the present study; Table S4: Primary antibodies used for Western blot.
