# Supplementary material for: Astaxanthin–Turmeric–Honeysuckle Extract Combination Attenuates DSS-Induced Colitis with Preservation of the Epithelial Barrier and Reduced NLRP3/Caspase-1/GSDMD Signaling
Source: Molecules. 2026 Sep 17;31(18):3303. doi: 10.3390/molecules31183303 (PMC13609881; doi:10.3390/molecules31183303)
Supplement: Supplementary file 1 [file molecules-31-03303-s001.zip › molecules-4488413-supplementary.pdf]

Supplementary Materials

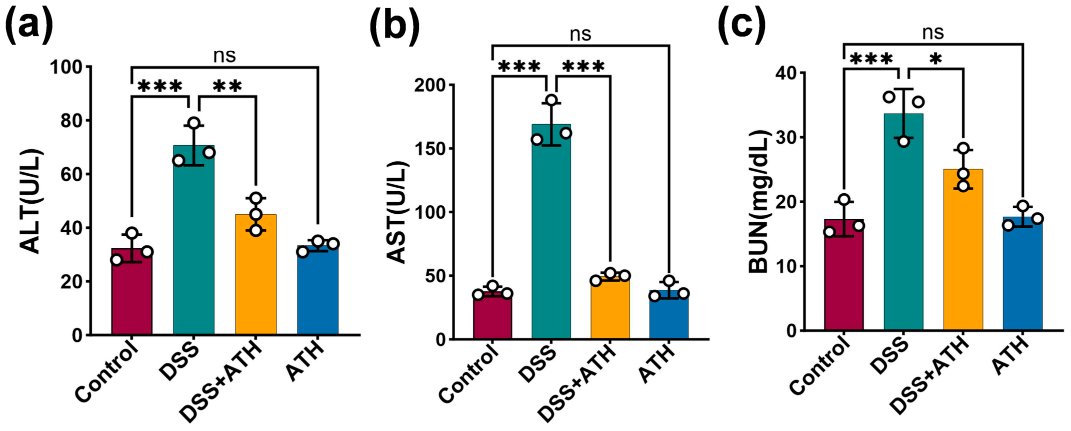

Figure S1. Effects of ATH on serum biochemical indices associated with hepatic and renal function in DSS-induced colitis. (a) Serum alanine aminotransferase (ALT) activity. (b) Serum aspartate aminotransferase (AST) activity. (c) Serum blood urea nitrogen (BUN) level. Data are presented as mean ± SD (n = 3 mice per group). * $P < 0.05$, ** $P < 0.01$, and *** $P < 0.001$ between the indicated groups; ns, not significant.

Table S1. Chemical characteristics and contents of major curcuminoids in the turmeric extract.

Major constituent	Subclass	Molecular formula	Molecular weight (g/mol)	Extract (mg/g)
Curcumin	diarylheptanoid	$C_{21}H_{20}O_6$	368.38	124.68 ± 6.69
Demethoxycurcumin	diarylheptanoid	$C_{20}H_{18}O_5$	338.35	60.32 ± 2.06
Bisdemethoxycurcumin	diarylheptanoid	$C_{19}H_{16}O_4$	308.33	38.91 ± 1.15

Table S2. Chemical characteristics and contents of major marker constituents in the honeysuckle extract.

Major constituent	Subclass	Molecular formula	Molecular weight (g/mol)	Extract (mg/g)
Chlorogenic acid	Mono-caffeoylquinic acid	$C_{16}H_{18}O_9$	354.31	16.38 ± 0.24
Isochlorogenic acid A	Dicaffeoylquinic acid	$C_{25}H_{24}O_{12}$	516.45	2.07 ± 0.08
Sweroside	Secoiridoid glycoside	$C_{16}H_{22}O_9$	358.34	1.06 ± 0.03
Secoxyloganin	Secoiridoid glycoside	$C_{17}H_{24}O_{11}$	404.37	1.87 ± 0.17
Morroniside	Iridoid glycoside	$C_{17}H_{26}O_{11}$	406.38	0.49 ± 0.07
Swertiamarin	Secoiridoid glycoside	$C_{16}H_{22}O_{10}$	374.34	0.35 ± 0.02
Rutin	Flavonol glycoside	$C_{27}H_{30}O_{16}$	610.52	1.15 ± 0.06
Luteolin-7-O-glucoside	Flavone glycoside	$C_{21}H_{20}O_{11}$	448.38	0.88 ± 0.09

Table S3. Composition and weight-based dosing of the ATH formulation used in the present study.

Component	Administered dose (mg/kg BW/day)	Mass ratio	Proportion of total ATH components (%, w/w)
Astaxanthin preparation	150	3	27.27
Turmeric extract	200	4	36.36
Honeysuckle extract	200	4	36.36
Total ATH components	550	11	100

Table S4. Primary antibodies used for Western blot.

Primary antibody	Manufacturer	Molecular weight (kDa)	Dilution
ZO-1	Nature Biosciences, Hangzhou, China	220	1:2000
Occludin	Nature Biosciences, Hangzhou, China	59	1:2000
NF-κB	Nature Biosciences, Hangzhou, China	65	1:2000
p-NF-κB	Nature Biosciences, Hangzhou, China	65	1:2000
NLRP3	Nature Biosciences, Hangzhou, China	118	1:2000
Caspase-1	Nature Biosciences, Hangzhou, China	45	1:2000
Cleaved Caspase-1	Nature Biosciences, Hangzhou, China	20	1:2000
N-GSDMD	Nature Biosciences, Hangzhou, China	35	1:2000
β-actin	Nature Biosciences, Hangzhou, China	42	1:5000
